# Identification of Prognostic and Predictive Biomarkers and Druggable Targets among 205 Antioxidant Genes in 21 Different Tumor Types via Data-Mining

**DOI:** 10.3390/pharmaceutics15020427

**Published:** 2023-01-28

**Authors:** Nadire Özenver, Thomas Efferth

**Affiliations:** 1 Department of Pharmaceutical Biology, Institute of Pharmaceutical and Biomedical Sciences, Johannes Gutenberg University, Staudinger Weg 5, 55128 Mainz, Germany; 2 Department of Pharmacognosy, Faculty of Pharmacy, Hacettepe University, Ankara 06100, Turkey

**Keywords:** drug resistance, Kaplan–Meier analysis, oxidative stress, prognostic value, survival analysis, the cancer genome atlas

## Abstract

(1) Background: Oxidative stress is crucial in carcinogenesis and the response of tumors to treatment. Antioxidant genes are important determinants of resistance to chemotherapy and radiotherapy. We hypothesized that genes involved in the oxidative stress response may be valuable as prognostic biomarkers for the survival of cancer patients and as druggable targets. (2) Methods: We mined the KM Plotter and TCGA Timer2.0 Cistrome databases and investigated 205 antioxidant genes in 21 different tumor types within the context of this investigation. (3) Results: Of 4347 calculations with Kaplan–Meier statistics, 84 revealed statistically significant correlations between high gene expression and worse overall survival (*p* < 0.05; false discovery rate ≤ 5%). The tumor types for which antioxidant gene expression was most frequently correlated with worse overall survival were renal clear cell carcinoma, renal papillary cell carcinoma, and hepatocellular carcinoma. Seventeen genes were clearly overexpressed in tumors compared to their corresponding normal tissues (*p* < 0.001), possibly qualifying them as druggable targets (i.e., *ALOX5*, *ALOX5AP*, *EPHX4*, *G6PD*, *GLRX3*, *GSS*, *PDIA4*, *PDIA6*, PRDX1, *SELENOH*, *SELENON*, *STIP1*, *TXNDC9*, *TXNDC12*, *TXNL1*, *TXNL4A*, and *TXNRD1*). (4) Conclusions: We concluded that a sub-set of antioxidant genes might serve as prognostic biomarkers for overall survival and as druggable targets. Renal and liver tumors may be the most suitable entities for this approach.

## 1. Introduction

Oxidative stress is widely recognized to be involved in many aging and disease processes, e.g., cancer, neurodegenerative diseases, chronic obstructive pulmonary disease, chronic kidney disease, etc. [1]. Reactive oxygen (ROS) and reactive nitrogen species (RNS) are involved in this process. ROS and RNS can damage cells through lesions in DNA, RNA, and proteins, as well as lipid peroxidation [2]. ROS-induced DNA damage contributes to carcinogenesis [3]. The human body has multiple antioxidant mechanisms and DNA repair mechanisms [4,5]. Despite the effectiveness of these defense mechanisms, persistent DNA damage occurs as the initial events of carcinogenesis. Antioxidant agents, including zinc, selenium, vitamins A, C, and E, and polyphenolic plant compounds are, therefore, considered promising for cancer prevention [6,7,8]. During carcinogenesis, the expression and activity of antioxidant proteins may be upregulated [9,10,11]. This carcinogenic deregulation does not lead to the protection of the body from ROS-induced damage; on the contrary, it leads to the protection of the tumor from ROS-inducing anticancer agents and the development of drug resistance [12,13,14,15]. A recently established therapeutic strategy aims to target and specifically inhibit cancer-related proteins, thereby effectively killing the tumor while sparing the normal tissue. Examples of clinically established targeted therapeutics are tyrosine kinase inhibitors [16,17,18] and therapeutic monoclonal antibodies [19,20,21].

Following the paradigm of targeted therapy, strategies can be developed to specif-ically address the upregulation of antioxidant proteins. The goal of inhibiting antioxidant genes and proteins in tumors should be to resensitize tumors to standard chemotherapy again. To approach this goal, however, we first need to know which genes and proteins are upregulated in tumors and are of prognostic importance for the survival of patients. Furthermore, it is necessary to analyze which tumor types are particularly suitable for such an approach using targeted antioxidant inhibitors. The aim of the present study was a systematic analysis of antioxidant genes that are overexpressed in tumors and are associated with an unfavorable survival prognosis. To this end, we chose the following strategy:

(1) Mining the literature, we identified 205 genes that are involved in antioxidant response. (2) Using the Kaplan–Meier plotter (https://kmplot.com/analysis/; accessed on 19 October 2022) [22,23], overall survival curves demonstrating the worse survival prognoses of these genes in 21 different tumor entities were identified. (3) Next, we compared the expression of these genes in tumors and corresponding normal tissues by using the Timer2.0 Cistrome tool of The Cancer Genome Atlas (https:/timer.cistrome.org) to identify genes that are significantly highly expressed in tumors, with the aim of delineating genes that may serve as druggable targets for targeted therapy in the future.

## 2. Materials and Methods

### 2.1. Kaplan–Meier Survival Analysis

Kaplan–Meier statistics are a well-established standard tool to calculate the probability of death based on diagnostic and molecular biomarkers (e.g., gene expression) or clinicopathological parameters (e.g., treatment, stage, and grade). In this investigation, we used the KM Plotter online tool (https://kmplot.com/analysis/) [22,23]. The KM Plotter is an online repository with data regarding mRNA, miRNA, protein expression, and DNA data based on diverse “-omics” technologies (RNA-Seq, proteomic data, etc.) from more than 30,000 samples of 21 tumor types. This database enables the identification of biomarkers for the survival of cancer patients. To cope with type I errors of multiple comparisons, we applied false discovery rate (FDR) corrections [24]. We included only Kaplan–Meier statistics with FDR rates ≤ 5%, indicating that not more than 5% of “declared” positive results were truly negative.

### 2.2. Comparative mRNA Expression in Tumor and Normal Samples

The Cancer Genome Atlas (TCGA) is a program driven by the National Cancer Institute, USA, that investigated more than 20,000 cancers and matched normal tissues from 33 cancer types for gene expressions, gene mutations, markers of genetic instability (rearrangements, deletions, and amplifications), and protein expressions. The Timer2.0 Cistrome tool compared the mRNA expression of genes in tumors and their matched normal tissues (https://timer.cistrome.org). In this investigation, we examined the cancerous and normal expression of genes that have been previously identified as candidate genes using Kaplan–Meier analyses.

## 3. Results

### 3.1. Assembling a List of Antioxidant Genes

As a first step, we mined the scientific literature for genes and proteins that are involved in antioxidant stress response. Based on a previous publication [25], we further screened the PubMed literature database to obtain an updated list of antioxidant genes. If identified genes belonged to a gene family with several other members, the other gene family members were also included in our list. A total of 205 genes/proteins were assembled. Their names, gene symbols, and functions are shown in Supplementary Table S1.

### 3.2. Correlation of mRNA Expression of Antioxidant Genes with Survival Prognosis of Patients

**Figure 1:**
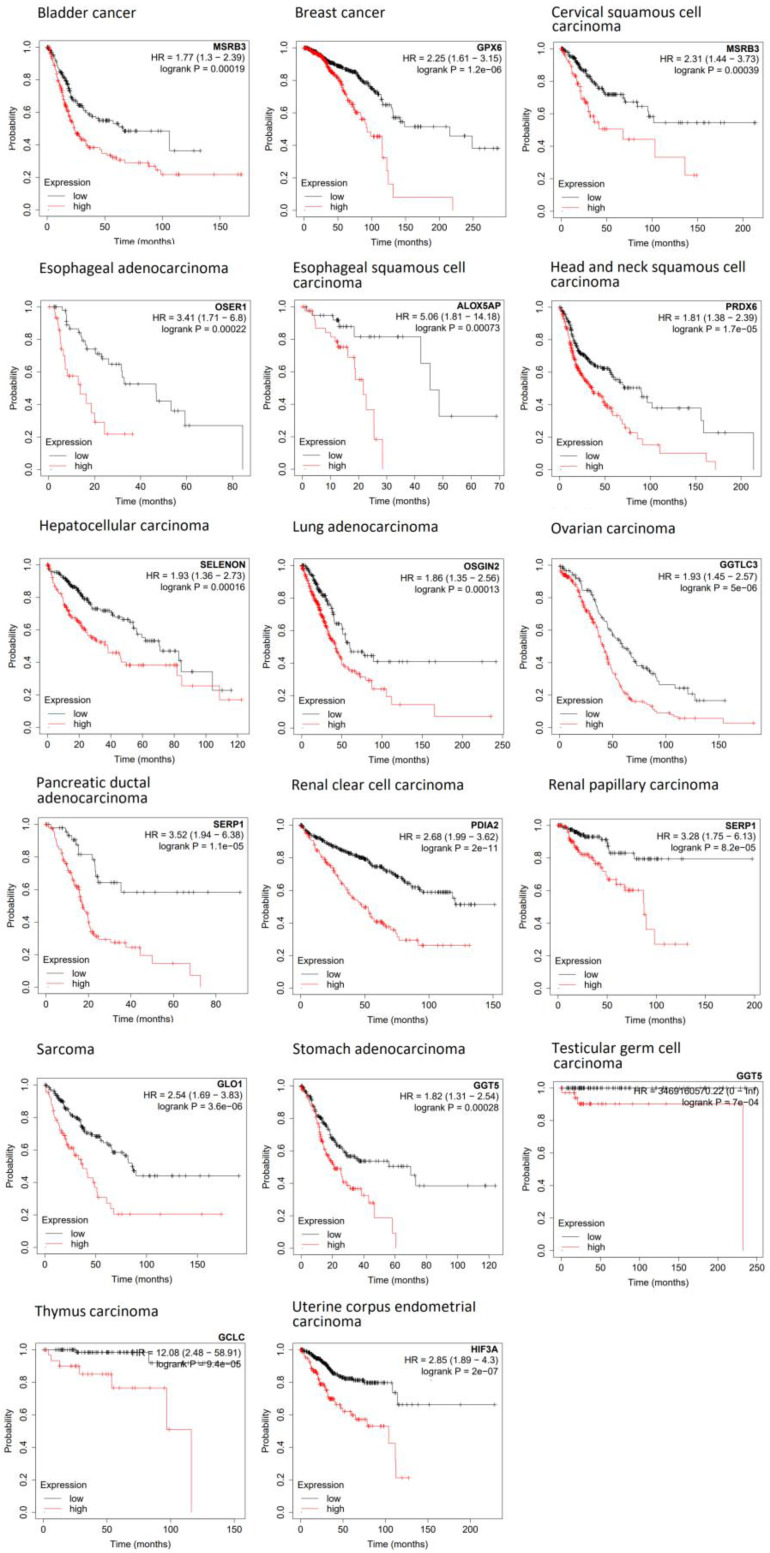
Kaplan–Meier statistics of overall survival for 17 tumor types.

Using Kaplan–Meier statistics, the mRNA expressions of these 205 genes were correlated with the overall survival times of patients. For this purpose, we took advantage of the KM Plotter database (https://kmplot.com/analysis/) that contains the mRNA sequencing data of 7489 individual tumors derived from 21 tumor types. We performed 4347 Kaplan–Meier calculations. Only 3 of 21 tumor types did not show statistically significant Kaplan–Meier statistics with any of the 205 genes (esophageal squamous cell carcinoma, lung squamous cell carcinoma, and thyroid carcinoma). In 17 tumor types, we obtained 845 calculations with a statistical significance of *p* < 0.05. Figure 1 shows representative examples of overall survival curves based on Kaplan–Meier statistics of each of these 17 tumor types.

**Figure 2:**
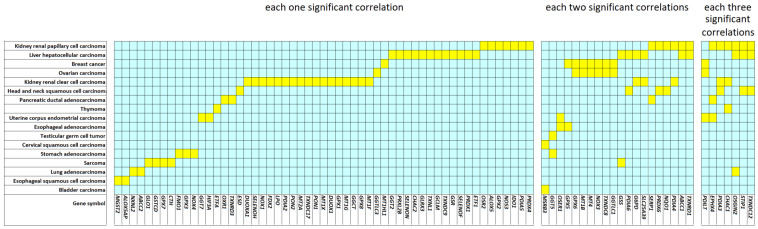
Color-coded plot of Kaplan–Meier statistics of 84 genes for 17 tumor types. Yellow indicates significant correlations between high gene expression in tumors and worse overall survival of patients. Blue indicates irrelevant outcomes.

To cope with the type I error problem of multiple significance calculations, we used a false discovery rate (FDR) of 5% as a threshold. As shown in Figure 2, 117 Kaplan–Meier calculations fulfilled this criterion. Out of all 205 genes investigated, only 84 displayed at least one significant survival statistic in one of the 17 tumor types. Of these, 55 were associated with patient survival of one tumor type, 22 with two tumor types, and 7 with three tumor types.

**Figure 3:**
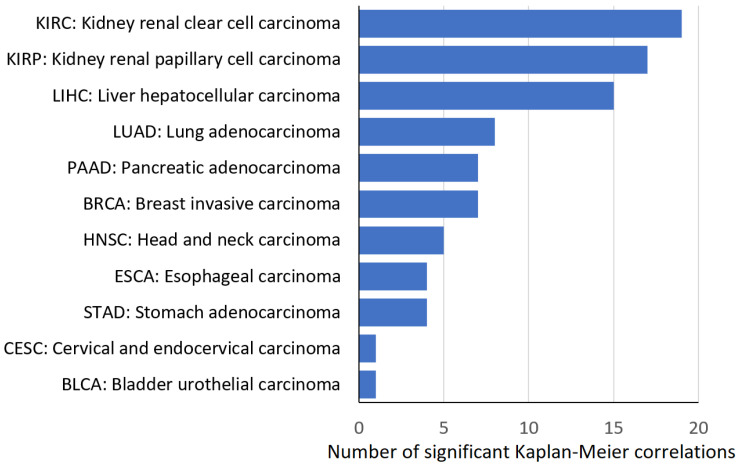
Number of significant Kaplan–Meier calculations for 11 tumor types.

We wanted to see in which tumor types antioxidant gene expression was most frequently correlated with worse overall survival. The three most frequently appearing tumor entities were renal clear cell carcinoma (KIRC), renal papillary cell carcinoma (KIRP), and hepatocellular carcinoma (LIHC), followed by lung adenocarcinoma (LUAD), breast cancer (BRCA), and pancreatic ductal adenocarcinoma (PAAD) (Figure 3).

After evaluating 7489 individual tumors, we focused on 84 genes and 6 tumor types (KIRC, KIRP, LIHC, LUAD, BRCA, and PAAD).

### 3.3. mRNA Expression of Antioxidant Genes in Tumor and Normal Tissues

**Figure 4:**
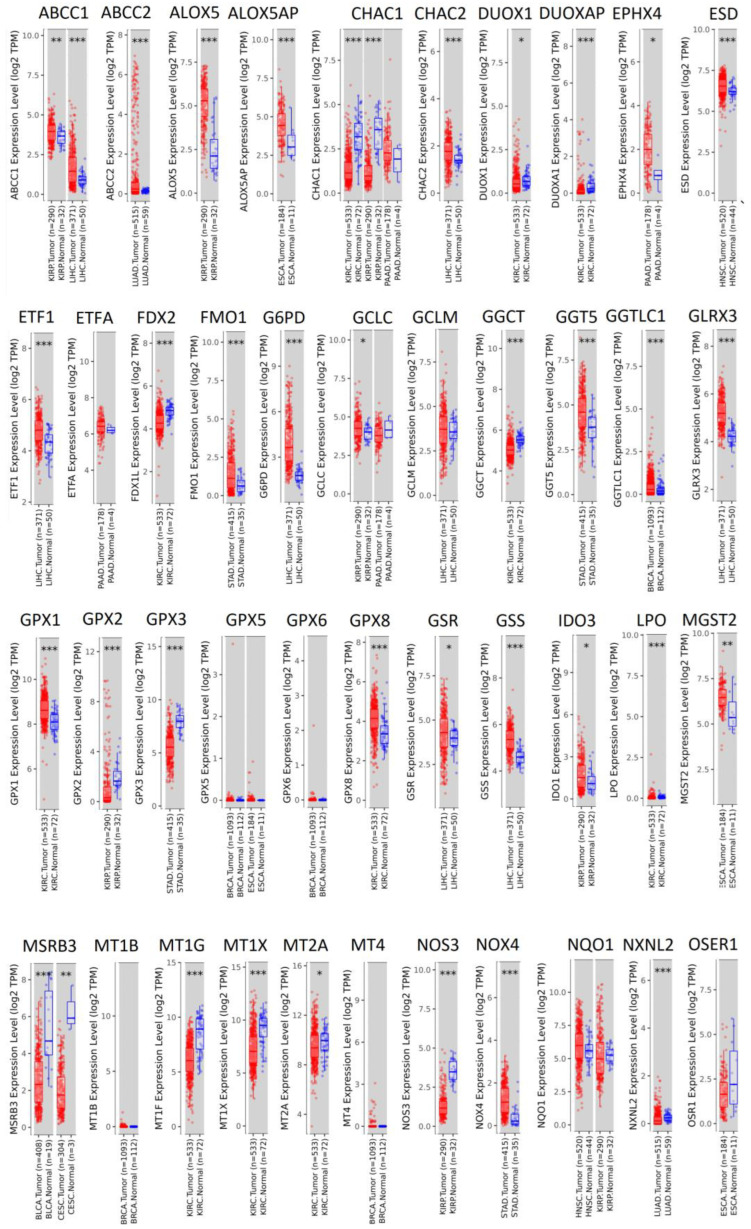

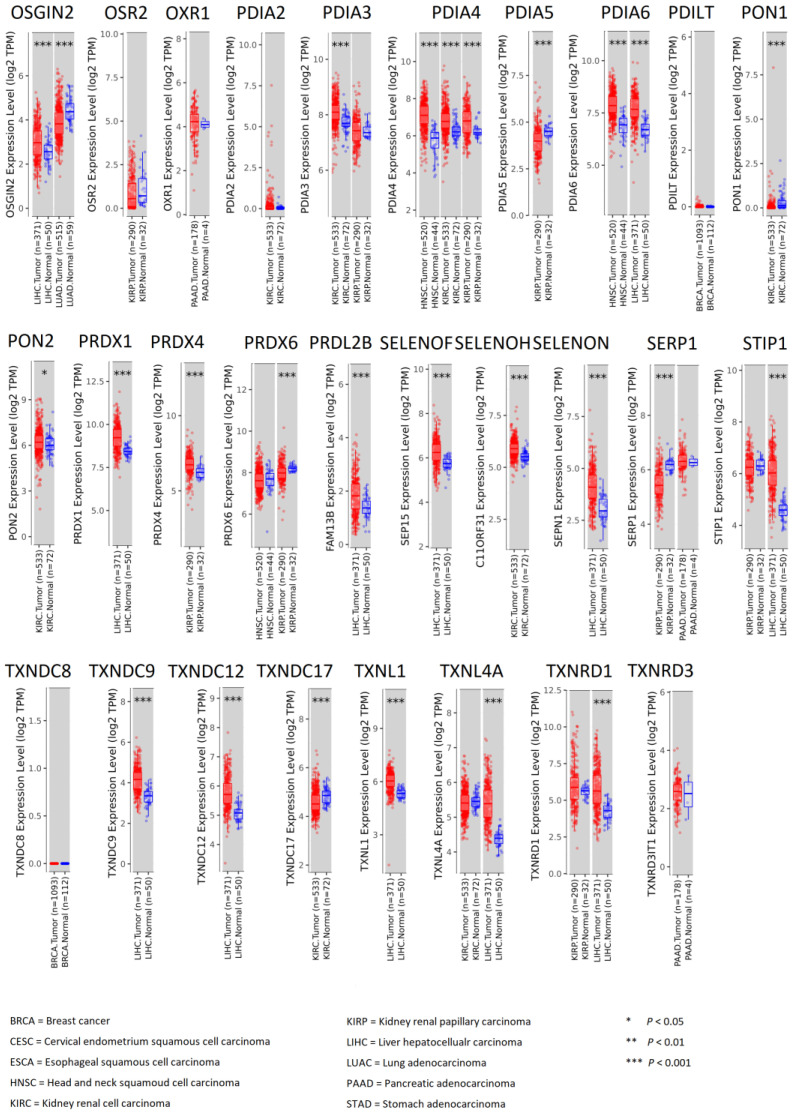
Comparative expression of 71 genes in tumors and corresponding normal tissues (part 1). Comparative expression of 71 genes in tumors and corresponding normal tissues (part 2).

Next, we compared the mRNA expression of these 84 genes not only in tumors, but also in the corresponding normal tissues to see whether or not the gene expression was tumor specific, i.e., more expressed in tumors than in normal tissues. For this reason, we mined the Timer2.0 Cistrome database of The Cancer Genome Atlas. We only included pairs of tumor and normal tissues where the corresponding expression values of normal tissues were deposited in the database; we excluded ovarian carcinoma, thymoma, testicular germ cell carcinoma, and uterine endometrial squamous cell carcinoma from further analysis. Thereby, the number of genes was narrowed down from 84 to 71. In 88 Kaplan–Meier statistical calculations from 71 genes, 43 (=49.9%), showed a statistically significant overexpression of the gene of interest compared to the corresponding normal tissue (Figure 4). In 21 calculations (=23.9%), the mRNA expression was not statistically different in tumor and normal tissues; in 24 (=27.3%), the mRNA expression in normal tissues was even higher than that of tumor tissues. Figure 4 shows three statistical levels (*p* < 0.05, <0.01, or < 0.001). Even if we focused only on those cases where the statistical level was *p* < 0.001, visual inspection revealed that in only 17 cases a clear distinction between mRNA expression was apparent (i.e., *ALOX5, ALOX5AP, EPHX4, G6PD, GLRX3, GSS, PDIA4* (in HNSC), *PDIA6, PRDX1, SELENOH, SELENON, STIP1* (in LIHC), *TXNDC9, TXNDC12, TXNL1, TXNL4A,* and *TXNRD1*).

In summary, we further downscaled the number of genes of interest to 17. 

## 4. Discussion

The basic concept of this investigation was to identify biomarkers and druggable targets from a large panel of oxidative stress response genes. In the past few years, a paradigm shift took place in cancer therapy from cytotoxic to targeted therapy [26,27,28,29]. This concept can be applied to cancer prevention as well as to cancer therapy. Although there is a plethora of compounds that have been described to act in a cancer-preventive manner because of their antioxidant characteristics [30,31], systematic searches for target-specific inhibitors of antioxidant proteins are rare. As antioxidant genes play an important role in the resistance of tumors to standard chemotherapy [14,32,33], inhibitors of antioxidant proteins may resensitize tumors to chemotherapeutic drugs.

We focused on genes with upregulated expressions and the worst survival prognoses. To identify potential biomarkers and targets for treatment, the overlap of genes with upregulated expression and relationship to prognosis represented a precondition. We did not consider the expression of genes without prognostic difference. To facilitate future drug development, we require overexpressed genes that are linked to short patient survival. If we inhibit such genes, the expectation is that, thereby, patient survival can be prolonged. Our manuscript describes the identification of these genes as a first step in the concept of individualized treatment with a combination of biomarker-driven diagnosis of antioxidant genes and subsequent treatment with targeted drugs against these genes and their encoded proteins.

This investigation represents a data-mining approach. Its first aim was to identify genes whose expression in tumors are associated with a worse overall patient survival prognosis. The second aim was to analyze these antioxidant genes for their potential suitability as druggable targets. This analysis compared the mRNA expression in tumors and normal tissues. Applying this approach identified 17 potential genes from the 205 genes analyzed.

Several thioredoxin-related genes appeared in our analysis (*TXNDC9*, *TXNDC12*, *TXNL1*, *TXNL4A*, and *TXNRD1*). Thioredoxins are electron-transferring oxidoreductases that counteract oxidative stress via thiol reduction and thereby contribute to redox homeostasis in cells [34,35,36]. The thioredoxin system is involved in tumors’ drug resistance [37]. It has been suggested that inhibitors may improve the response of tumors to anticancer drugs [37,38]. Regarding genes in this analysis, *TXNDC9* was reported to be upregulated upon oxaliplatin treatment and may confer oxaliplatin resistance in colorectal adenocarcinoma cells [39]. *TXNRD1* was a significant predictor of poor treatment outcome in non-small cell lung cancer and was correlated with shorter disease-free survival [40]. *TXNRD1* was proposed as a biomarker to monitor therapeutic efficacy for liver cancer [41]. Interestingly, *TXNRD1* has been inhibited by numerous electrophilic compounds [42]. It can be concluded that thioredoxin-related proteins may serve as treatment targets to improve standard cancer chemotherapy.

Similar to thioredoxin, periredoxins (*PRDX*) are reactive oxygen species (ROS) scavengers that maintain redox homeostasis. Higher *PRDX1* expression in tumors than in corresponding normal tissues has been observed in cervical carcinoma and Burkitt lymphoma [43,44,45]. Hypermethylation silenced the *PRDX1* promoter and sensitized brain tumors to temozolomide and ionizing radiation [46] *PRDX1* overexpression in glioma cells enhanced resistance to bis-chloroethyl nitrosurea (BCNU) [47]. In ovarian carcinoma, high *PDRX1* expression was associated with poor response to chemotherapy and lower 5-year disease-free survival [48]. Interestingly, the naturally occurring small-molecular peptidomimetic SK053 targeted *PRDX1* and *PRDX2*, leading to cell cycle arrest and apoptosis in Burkitt lymphoma cells [44]. These data indicate that *PRDX1* inhibition might be a valuable strategy to reduce oxidative stress and increase chemosensitivity in cancer cells.

The expression of protein disulfide isomerase 4 (*PDIA4*) was correlated to docetaxel resistance by inhibiting apoptosis and activating the Akt-signaling pathway [49]. Furthermore, *PDIA6* upregulation enhanced cisplatin resistance in gastric cancer cells [50]. Inhibitors of *PDIA4* and *PDIA6* deserve further investigation as chemosensitizers.

Stress-induced phosphoprotein 1 (*STIP1*) is a co-chaperone involved in the transfer of damaged proteins to the heat shock proteins *HSP70* and *HSP90* [51]. In advanced bladder carcinoma, high *STIP1* expression significantly correlated with worse overall survival and chemotherapeutic pretreatment with a cisplatin-based regimen [52]. Whether *STIP1* has potential as biomarker for survival and as predictive marker for drug resistance in other tumor types warrants further investigation.

While glutathione and enzymes involved in the glutathione redox cycle have been intensively discussed regarding their role in anticancer drug resistance [15,53,54], the role of glutathione synthetase (GSS) in chemoresistance is largely unknown. A single nucleotide polymorphism study in 903 small cell lung cancer patients revealed that the rs725521 polymorphism in GSS was significantly correlated with response to platin-based chemotherapy [55]. The relevance of GSS for drug resistance should be clarified in future studies.

The antioxidant and chemopreventive features of selenium have been widely investigated for several diseases, including cancer [56,57,58]. Selenium compounds also induced apoptosis and prevented the development of cisplatin resistance [59,60,61]. The role of *SELENOF* and *SELENOH* genes, which appeared in this investigation as candidate biomarkers, is unknown from the literature, but deserves further investigation.

Glucose 6-phosphate dehydrogenase (*G6PD*) is a key enzyme in the pentose phosphate cycle. *G6PD* oxidizes glucose-6-phosphate and reduces NADP+. A deficiency in this enzyme is associated with the most inherited diseases, affecting approximately 5% of the world’s population. Patients suffering from *G6PD* deficiency are hypersensitive to oxidative stress and susceptible to hemolytic anemias [62,63,64,65]. Leukocytes of *G6PD*-deficient patients are more prone, in vitro, to induce apoptosis upon exposure to daunorubicin, dexamethasone, ionizing radiation, and UV radiation compared to healthy leukocytes [66,67,68]. Data for *G6PD*-deficient cancer patients are rare, either because patients are not routinely tested for their *G6PD* status or because anticancer chemotherapy remains without negative symptoms in these patients [69,70]. Therefore, the value of *G6PD* inhibitors to sensitize *G6PD*-positive cancer patients to chemotherapy remains elusive.

There are contrary results regarding the role of arachidonate 5-lipoxygenase (*ALOX5*) for drug resistance. *ALOX5* overexpression in gastric cancer cells was associated with reduced drug activity, and genetic or pharmacological *ALOX5* inhibition increased drug efficacy [71]. On the other hand, *ALOX5* overexpression in acute myeloid leukemia increased sensitivity to chemotherapy [72]. A conclusion regarding the feasibility of *ALOX5* inhibitors as possible chemosensitizers cannot yet be drawn.

In addition to genes and proteins that may serve as prognostic and predictive biomarkers, it is important to clarify which tumor types are the most susceptible to inhibition of antioxidant genes/proteins. We found that the overall survival of renal clear cells carcinoma (KIRC), renal papillary cell carcinoma (KIRP), and hepatocellular carcinoma (LIHC) was most frequently associated with high expression of antioxidant genes. This indicates that these tumor entities may be more suitable for chemosensitization via inhibition of antioxidant genes than the other tumor types investigated. Interestingly, these tumor types are known to be rather chemoresistant [73,74,75,76,77,78]. Hence, chemosensitization strategies may be attractive to improve treatment outcomes.

Renal clear cell carcinoma is not usually treated with cytotoxic chemotherapy because of its ineffectiveness. Surgery, immunotherapy, and targeted therapy with tyrosine kinase inhibitors are applied with modest success [73,74,79]. The situation in renal papillary cell carcinoma is not fundamentally different [75,80]. Hepatocellular carcinoma can be treated with monoclonal antibodies and tyrosine kinase inhibitors [76,77,78,81]. Chemotherapy with doxorubicin and cisplatin can be applied with limited success. The 5-year overall survival rates are between 20 and 50%. Therefore, investigating possibilities for chemosensitization to treat these three tumor entities using inhibitors of antioxidant genes as discussed above may be meritorious in light of their currently limited cure rates.

## 5. Conclusions

We identified a panel of antioxidant genes, using our transcriptome-wide RNA-sequencing data-mining approach, as possible biomarkers with prognostic value for overall survival and predictive value for poor response to chemotherapy. Additionally, these genes/proteins may serve as druggable targets in the search for specific inhibitors of these genes/proteins to resensitize tumors for standard chemotherapy. In future studies their potential as biomarkers should be further validated and specific small molecule inhibitors should be screened as chemosensitizers to overcome drug resistance. Researchers should discuss and interpret their results from the perspective of previous studies and working hypotheses. The findings and the implications thereof should be discussed in the broadest context possible. Future research directions may also be highlighted.

## Data Availability

Not applicable.

## Supplementary Materials

The following are available online at https://www.mdpi.com/article/10.3390/pharmaceutics15020427/s1, Table S1: Symbols, names, and functions of genes involved in antioxidant response.
