# Supplementary material for: Identification of Prognostic and Predictive Biomarkers and Druggable Targets among 205 Antioxidant Genes in 21 Different Tumor Types via Data-Mining
Source: Pharmaceutics. 2023 Jan 28;15(2):427. doi: 10.3390/pharmaceutics15020427 (PMC9959161; doi:10.3390/pharmaceutics15020427)
Supplement: Supplementary file 1 [file pharmaceutics-15-00427-s001.zip › pharmaceutics-2130017-Supplemenatary table 1.pdf]

Table S1. Symbols, names, and functions of genes involved in antioxidant response. (The data are retrieved from https://www.genecards.org and https://www.ncbi.nlm.nih.gov/gene databases.)

Symbol	Name	Function
ABCC1	ATP-binding cassette subfamily C member 1	Cytosolic export of organic anions and drugs, drug resistance, inflammatory response
ABCC2	ATP-binding cassette subfamily C member 2	Active transport of drugs, toxicants, and endogenous compounds across cell membranes, drug resistance
ACO1	Aconitase 1	Isomerization of citrate to isocitrate.
ACO2	Aconitase 2	Isomerization of citrate to isocitrate.
ALOX5	Arachidonate 5-lipoxygenase	Oxygenation of arachidonate to 5-hydroperoxyeicosatetraenoate, inflammatory processes, dendritic cell migration, antioxidative wound healing.
ALOX5AP	Arachidonate 5-lipoxygenase activating protein	Leukotriene biosynthesis by ALOX5, regulation of redox homeostasis.
AOX1	Aldehyde oxidase 1	source of superoxide and peroxide generation via the one-electron reduction.
ATOX1	Antioxidant 1 copper chaperone	Deliver cytosolic copper to copper ATPase proteins, antioxidant defense.
CAT	Catalase	Protection from toxic hydrogen peroxide effects.
CCS	Copper chaperone for superoxide dismutase	Delivers copper to copper zinc superoxide dismutase,
CHAC1	ChaC glutathione specific γ-glutamylcyclotransferase 1	Interacts with glutathione, pro-apoptotic component of the unfolded protein response pathway.
CHAC2	ChaC glutathione specific γ-glutamylcyclotransferase 2	Interacts with glutathione, cleavage of glutathione into 5-oxo-L-proline and a Cys-Gly dipeptide.
CP	Ceruloplasmin	Iron and copper homeostasis, ferroxidase, amine oxidase, and superoxide dismutase activities.
CTH	Cystathionine γ-lyase	Biosynthesis of glutathione.
DUOX1	Dual oxidase 1	Generates hydrogen peroxide.
DUOX2	Dual oxidase 2	Generates hydrogen peroxide.
DUOXA1	Dual oxidase maturation factor 1	Maturation and transport of functional DUOX1 from the endoplasmic reticulum to the plasma membrane.
DUOXA2	Dual oxidase maturation factor 2	Maturation and transport of functional DUOX2 from the endoplasmic reticulum to the plasma membrane.
EPHX1	Epoxide hydrolase 1	Hydrolysis of arene and aliphatic epoxides to less reactive and more water soluble dihydrodiols.

**Symbol**	**Name**	**Function**
EPHX2	Epoxide hydrolase 2	Degradation of toxic epoxides.
EPHX3	Epoxide hydrolase 3	Hydrolysis of epoxide-containing fatty acids.
EPHX4	Epoxide hydrolase 4	Hydrolysis and protein binding.
ESD	Esterase D/formylglutathione hydrolase	Hydrolysis of S-formylglutathione to reduced glutathione and formate.
ETF1	Eukaryotic translation termination factor 1	SHFL-mediated translation termination inhibiting programmed ribosomal frameshifting (-1PRF) of viral and cellular mRNA.
ETFA	Electron transfer flavoprotein subunit α	Electron transfer to the main mitochondrial respiratory chain via ETF-ubiquinone oxidoreductase, normal mitochondrial fatty acid oxidation and normal amino acid metabolism.
ETFB	Electron transfer flavoprotein subunit β	See *ETFA*.
ETFDH	Electron transfer flavoprotein dehydrogenase	Electron acceptor from ETF, reduces ubiquinone.
ETFRF1	Electron transfer flavoprotein regulatory factor 1	Electron transfer by removal of flavin from ETF holoenzyme.
FDX1	Ferredoxin 1	Electron transport intermediate for mitochondrial cytochromes P450.
FDX2	Ferredoxin 2	Essential for heme A and Fe/S protein biosynthesis.
FDXACB1	Ferredoxin-fold anticodon binding domain containing 1	Protein binding, rRNA (uridine-N3-)-methyltransferase activity.
FDXR	Ferredoxin reductase	First electron transfer protein in all the mitochondrial P450 systems.
FMO1	Flavin containing dimethylaniline monoxygenase 1	Oxidative metabolism of xenobiotics such as drugs and pesticides.
FMO2	Flavin containing dimethylaniline monoxygenase 2	See *FMO1*.
FMO3	Flavin containing dimethylaniline monoxygenase 3	Oxygenation of nitrogen- and sulfur-containing compounds including drugs and dietary compounds.
FMO4	Flavin containing dimethylaniline monoxygenase 4	See *FMO1*.
FMO5	Flavin containing dimethylaniline monoxygenase 5	Oxidation of nucleophilic nitrogen, sulfur, and phosphorus atoms in many compounds.
G6PD	Glucose-6-phosphate dehydrogenase	Rate-limiting step of the oxidative pentose-phosphate pathway, provides NADPH and pentose phosphates for fatty acid and nucleic acid synthesis.
GCLC	γ-Cysteine ligase catalytic subunit	ATP-dependent ligation of L-glutamate and L-cysteine, first and rate-limiting step in glutathione biosynthesis.

Symbol	Name	Function
GCLM	γ-Cysteine ligase modifier subunit	γ-Glutamyl cysteine synthetase, first step of GSH synthesis, γ-glutamyl cycle.
GGCT	γ-Glutamylcyclotransferase	Formation of 5-oxoproline from γ-glutamyl dipeptides, role in glutathione homeostasis.
GGT1	γ-Glutamyltransferase 1	Glutathione metabolism, releases free glutamate and the dipeptide cysteinyl-glycine.
GGT2	γ-Glutamyltransferase 2	γ-Glutamyl cycle.
GGT5	γ-Glutamyltransferase 5	Cleaves γ-glutamyl peptide bond of glutathione and glutathione-S-conjugates.
GGT6	γ-Glutamyltransferase 6	Hydrolyzes and transfers γ-glutamyl moieties from glutathione and other γ-glutamyl compounds to acceptors.
GGT7	γ-Glutamyltransferase 7	See GGT6.
GGTLC1	γ-Glutamyltransferase light chain 1	Glutathione hydrolysis
GGTLC2	γ-Glutamyltransferase light chain 2	See *GGTLC1.*
GGTLC3	γ-Glutamyltransferase light chain 3	See *GGTLC1.*
GLO1	Glyoxalase I	Conversion of hemimercaptal to S-lactoylglutathione.
GLOD5	Glyoxalase domain containing 5	Platelet volume and platelet count.
GLRX	Glutaredoxin	Glutathione-disulfide oxidoreductase.
GLRX2	Glutaredoxin 2	Glutathione-dependent hydrogen donor in cellular redox reactions.
GLRX3	Glutaredoxin 3	Required for hemoglobin maturation.
GLRX5	Glutaredoxin 5	Monothiol glutaredoxin involved in mitochondrial iron-sulfur (Fe/S) cluster transfer, required for hemoglobin synthesis.
GPX1	Glutathione peroxidase 1	Protects the hemoglobin in erythrocytes from oxidative breakdown, role in the arachidonic acid metabolism.
GPX2	Glutathione peroxidase 2	Protectiong from the toxicity of ingested organic hydroperoxides.
GPX3	Glutathione peroxidase 3	Protection from oxidative damage by reduction of hydrogen peroxide, lipid peroxides and organic hydroperoxide by glutathione.
GPX4	Glutathione peroxidase 4	Protection from toxicity of lipid hydroperoxides.
GPX5	Glutathione peroxidase 5	Protection from oxidative damage by reduction of hydrogen peroxide, lipid peroxides and organic hydroperoxide by glutathione.
GPX6	Glutathione peroxidase 6	Catalytic activity.
GPX7	Glutathione peroxidase 7	Protection of esophageal epithelia from hydrogen peroxide-induced oxidative stress. Suppression of acidic bile acid-induced reactive oxygen species and protection from oxidative DNA damage and DNA double-strand breaks.

Symbol	Name	Function
GPX8	Glutathione peroxidase 8	Catalytic activity.
GSR	Glutathione reductase	Maintains high cytosolic levels of reduced glutathione.
GSS	Glutathione synthetase	ATP-dependent production of glutathione from γ-glutamylcysteine and glycine.
GSTA1	Glutathione S-transferase α1	Formation of glutathione conjugates of prostaglandin A2 (PGA2) and prostaglandin J2 (PGJ2).
GSTA2	Glutathione S-transferase α2	Conjugation of reduced glutathione to exogenous and endogenous hydrophobic electrophiles.
GSTA3	Glutathione S-transferase α3	See *GSTA2*.
GSTA4	Glutathione S-transferase α4	See *GSTA2*.
GSTA5	Glutathione S-transferase α5	See *GSTA2*.
GSTCD	Glutathione S-transferase C-terminal domain containing	Molecular function, protein binding.
GSTK1	Glutathione S-transferase κ1	The conjugation of glutathione to exogenous and endogenous compounds.
GSTM1	Glutathione S-transferase μ1	Conjugation of reduced glutathione to a wide number of exogenous and endogenous hydrophobic electrophiles.
GSTM2	Glutathione S-transferase μ2	See *GSTM1*.
GSTM3	Glutathione S-transferase μ3	See *GSTM1*.
GSTM4	Glutathione S-transferase μ4	See *GSTM1*.
GSTM5	Glutathione S-transferase μ5	See *GSTM1*.
GSTO1	Glutathione S-transferase ω1	Glutathione-dependent thiol transferase and dehydroascorbate reductase.
GSTO2	Glutathione S-transferase ω2	Glutathione-dependent thiol transferase activity.
GSTP1	Glutathione S-transferase π1	Conjugation of reduced glutathione to a wide number of exogenous and endogenous hydrophobic electrophiles.
GSTT1	Glutathione S-transferase θ1	See *GSTP1*.
GSTT2	Glutathione S-transferase θ2	See *GSTP1*.
GSTT2B	Glutathione S-transferase θ2B	See *GSTP1*.
GSTT4	Glutathione S-transferase θ4	See *GSTP1*.
GSTZ1	Glutathione S-transferase ζ1	Glutathione-conjugating activity, glutathione peroxidase activity.
HAGH	Hydroxyacylglutathione hydrolase	Hydrolysis of S-D-lactoyl-glutathione to form glutathione and D-lactic acid.
HIF1A	Hypoxia-inducible factor 1, α subunit	Master regulator of homeostatic response to hypoxia by activating gene transcription.

Symbol	Name	Function
HIF1AN	Hypoxia inducible factor 1 subunit α inhibitor	Negative regulation of Notch signaling pathway and transcription from RNA polymerase II promoter in response to hypoxia; protein hydroxylation.
HIF3A	Hypoxia inducible factor 3 subunit α	Regulation of adaptive responses to hypoxia.
HMOX1	Heme oxygenase 1	Heme catabolism, cleaves heme to form biliverdin.
HMOX2	Heme oxygenase 2	See *HMOX2*.
HYOU1	Hypoxia up-regulated 1	Protein folding and secretion in the ER.
IDO1	Indoleamine 2,3-dioxygenase 1	Antimicrobial and antitumor defense, neuropathology, immunoregulation, and antioxidant activity.
IDO2	Indoleamine 2,3-dioxygenase 2	First and rate limiting step of the catabolism of tryptophan along the kynurenine pathway, immune regulation.
KEAP1	Kelch-like ECH associated protein 1	Response to oxidative stress.
LANCL1	LanC-like glutathione S-transferase 1	Conjugation of the glutathione (GSH) to artificial substrates 1-chloro-2,4-dinitrobenzene (CDNB) and p-nitrophenyl acetate. Mitigates neuronal oxidative stress during postnatal development and in response to oxidative stresses probably through GSH antioxidant defense mechanism.
LANCL2	LanC-like glutathione S-transferase 2	Binding of phosphatidylinositol-3-phosphate, phosphatidylinositol-4-phosphate, and phosphatidylinositol-5-phosphate, negative regulation of transcription and positive regulation of abscisic acid-activated signaling pathway.
LANCL3	LanC-like glutathione S-transferase 3	Carbohydrate metabolism.
LPO	Lactoperoxidase	Conversion of thiocyanate (SCN(-)) into antimicrobial agent hypothiocyanous acid (OSCN(-)) in the presence of H_2O_2, maintaining cellular H_2O_2 levels, protecting cells from H_2O_2-caused injuries and inflammation.
MAOA	Monoamine oxidase A	Oxidative deamination of neurotransmitters with concomitant reduction of oxygen to H_2O_2 in the CNS and peripheral tissues.
MAOB	Monoamine oxidase B	See *MAOA*.
MGST1	Microsomal glutathione S-transferase 1	Conjugation of reduced glutathione to exogenous and endogenous hydrophobic electrophiles.

Symbol	Name	Function
MGST2	Microsomal glutathione S-transferase 2	Glutathione-dependent reduction of lipid hydroperoxides, such as 5-HPETE, glutathione transfer to xenobiotic electrophiles, involved in oxidative DNA damage induced by ER stress and anticancer agents.
MGST3	Microsomal glutathione S-transferase 3	Oxydation of hydroxy-fatty acids, Conjugation of a reduced glutathione to leukotriene A4
MPO	Myeloperoxidase	Major component of neutrophil azurophilic granules.
MSRA	Methionine sulfoxide reductase A	Repair of oxidatively damaged proteins.
MSRB1	Methionine sulfoxide reductase B1	Protects proteins from oxidative stress by the reduction of methionine-R-sulfoxides to methionines.
MSRB2	Methionine sulfoxide reductase B2	Mitochondrial Peptide-methionine (R)-S-oxide reductase, actin filament polymerization and protein repair.
MSRB3	Methionine sulfoxide reductase B3	Reduction of methionine sulfoxide to methionine.
MT1A	Metallothionein 1A	Protects against hydroxyl free radicals, homeostatic control of cellular metals, detoxification of heavy metals.
MT1B	Metallothionein 1B	Binds heavy metals and protects against their toxicity.
MT1E	Metallothionein 1E	Involved in cellular response to cadmium ion and cellular response to zinc ion.
MT1F	Metallothionein 1F	Enables zinc ion binding activity. Involved in cellular response to cadmium ion. Located in cytoplasm and nucleus.
MT1G	Metallothionein 1G	Involved in cellular response to metal ion; cellular response to vascular endothelial growth factor stimulus; and negative regulation of growth.
MT1H	Metallothionein 1H	Involved in cellular response to cadmium ion and cellular response to zinc ion.
MT1HL1	Metallothionein 1H Like 1	Binds various heavy metals.
MT1M	Metallothionein 1M	Binds various heavy metals.
MT1X	Metallothionein 1X	Binds various heavy metals.
MT2A	Metallothionein 2A	Act as anti-oxidants, protect against hydroxyl free radicals,<br>Important in homeostatic control of metal ions in the cell, Play a role in detoxification of heavy metals.
MT3	Metallothionein 3	Zinc and copper homeostasis.

Symbol	Name	Function
MT4	Metallothionein 4	Cellular response to metal ions, cellular zinc ion homeostasis, and detoxification of copper ion.
NFE2L2	NFE2-like BZIP transcription factor 2	Key role in response to oxidative stress.
NIT1	Deaminated glutathione amidase	Role in cell growth and apoptosis: loss of expression promotes cell growth, resistance to DNA damage stress and increased incidence to NMBA-induced tumors.
NOA1	Nitric oxide-associated 1	Regulation of mitochondrial protein translation and respiration, role in mitochondria-mediated cell death.
NOS1	Nitric oxide synthase 1	Production of the messenger molecule nitric oxide (NO).
NOS1AP	Nitric oxide synthase 1 adaptor protein	Regulation of neuronal NO synthesis.
NOS2	Nitric oxide synthase 2	See *NOS1*.
NOS3	Nitric oxide synthase 3	See *NOS1*.
NOSIP	Nitric oxide synthase interacting protein	Negative regulation of NO production.
NOSTRIN	Nitric oxide synthase trafficking	Decreases NOS3 activity by translocating it away from the plasma membrane.
NOX1	NADPH oxidase 1	Regulation of cellular pH, generates superoxide, and conducts H^+ ions as part of its electron transport mechanism.
NOX2	NADPH oxidase 2	Superoxide-generating NAD(P)H oxidase.
NOX3	NADPH oxidase 3	Produces superoxide.
NOX4	NADPH oxidase 4	See *NOX3*.
NOX5	NADPH oxidase 5	Endothelial ROS generation, proliferation and angiogenesis, and endothelial response to thrombin.
NOXO1	NADPH oxidase organizer 1	Potentiates the superoxide-generating activity of NOX1 and NOX3.
NQO1	NAD(P)H quinone dehydrogenase 1	Regulates cellular redox state primarily through quinone detoxification.
NQO2	N-Ribosyldihydronicotinamide:quinone reductase 2	Quinone reductase, vitamin K-dependent γ-carboxylation of glutamate residues in prothrombin synthesis.
NXNL1	Nucleoredoxin-like 1	Role in retinal cone photoreceptor survival.
NXNL2	Nucleoredoxin-like 2	Maintenance of function and viability of sensory neurons, including photoreceptors and olfactory neurons.
OSER1	Oxidative stress responsive serine-rich 1	Response to hydrogen peroxide.
OSGIN1	Oxidative stress-induced growth inhibitor 1	Regulates differentiation and proliferation

Symbol	Name	Function
OSGIN2	Oxidative stress-induced growth inhibitor 2	Involved in meiosis or the maturation of germ cells.
OSR1	Odd-skipped related transcription factor 1	Regulation of embryonic heart and urogenital development.
OSR2	Odd-skipped related transciption factor 2	Binding of RNA polymerase II transcription regulatory region sequence-specific DNA.
OXR1	Oxidation resistance 1	May be involved in protection from oxidative damage.
OXSR1	Oxidative stress responsive kinase 1	Phosphorylates RELL1, RELL2 and RELT, PAK1, PLSCR1.
PDIA2	Protein disulfide isomerase A2	Binds estrogen, modulates estrogen functions in the pancreas.
PDIA3	Protein disulfide isomerase A3	Formation, isomerization, and reduction or oxidation of disulfide bonds.
PDIA4	Protein disulfide isomerase A4	Rearrangement of -S-S- bonds in proteins.
PDIA5	Protein disulfide isomerase A5	Oxidoreductase.
PDIA6	Protein disulfide isomerase A6	Negative regulation of the unfolded protein response. Platelet aggregation and activation.
PDILT	Protein disulfide isomerase-like, testis expressed	Redox-inactive chaperone in spermatogenesis.
PON1	Paraoxonase 1	Hydrolyzing organophosphate substrates, lactones, and aromatic carboxylic acid esters. Protection of LDL against oxidative modification and consequent atheroma formation.
PON2	Paraoxonase 2	Hydrolyzes lactones and aromatic carboxylic acid esters. Aas antioxidant activity. Prevents and reverses LDL lipid peroxidation, and inhibits MM-LDL to induce monocyte chemotaxis.
PON3	Paraoxonase 3	Hydrolyzes lactones (statin prodrugs).
PRDX1	Peroxiredoxin 1	Reduction of H_2O_2 and organic hydroperoxides to water and alcohols.<br>Protection from oxidative stress.
PRDX2	Peroxiredoxin 2	See *PRDX1*.
PRDX3	Peroxiredoxin 3	See *PRDX1*.
PRDX4	Peroxiredoxin 4	See *PRDX1*. Sensor of H_2O_2-mediated signaling.
PRDX5	Peroxiredoxin 5	See *PRDX5*.
PRDX6	Peroxiredoxin 6	See *PRDX1*.
PRXL2A	Peroxiredoxin-like 2A	Cellular redox regulation. Antioxidant.
PRXL2B	Peroxiredoxin-like 2B	Reduction of prostaglandin-ethanolamide H(2) (prostamide H(2)) to prostamide F(2alpha) with NADPH as proton donor.

Symbol	Name	Function
PRXL2C	Peroxiredoxin-like 2C	Regulates ERK1/2 signaling and AKT1 activation leading to HIF1A up-regulation and enhanced glycolysis.
SELENBP1	Selenium-binding protein 1	Sensing of reactive xenobiotics in the cytoplasm. Intra-Golgi protein transport.
SELENOF	Selenoprotein F	Redox reactions associated with disulfide bond formation.
SELENOH	Selenoprotein H	Redox-related processes.
SELENOI	Selenoprotein I	Synthesis of several PE species and ether-linked phospholipids.
SELENOK	Selenoprotein K	Protects from oxidative stress if overexpressed in cardiomyocytes.
SELENOM	Selenoprotein M	Thiol-disulfide oxidoreductase participating in disulfide bond formation.
SELENON	Selenoprotein N	Protection from oxidative stress. Regulation of redox-related calcium homeostasis. Protecting the calcium pump ATP2A2 against the oxidoreductase ERO1A-mediated oxidative damage.
SELENOO	Selenoprotein O	Redox-active mitochondrial selenoprotein interacting with redox target proteins.
SELENOP	Selenoprotein P	Extracellular antioxidant defense and transport properties of selenium.
SELENOS	Selenoprotein S	Degradation of misfolded ER luminal proteins.
SELENOT	Selenoprotein T	Protects dopaminergic neurons against oxidative stress and cell death.
SELENOV	Selenoprotein V	Redox-related processes.
SELENOW	Selenoprotein W	Glutathione (GSH)-dependent antioxidant.
SERP1	Stress Associated Endoplasmic Reticulum Protein 1	Glycosylation of target proteins after ER stress.
SERP2	Stress Associated Endoplasmic Reticulum Protein Family Member 2	May interact with target proteins during translocation into the lumen of the endoplasmic reticulum,<br>May protect unfolded target proteins against degradation and facilitate correct glycosylation.
SLC25A39	Solute Carrier Family 25 Member 39	Mitochondrial transporter required for glutathione import into mitochondria, Glutathione, which plays key roles in oxidative metabolism, is produced exclusively in the cytosol and is imported in many organelles.

Symbol	Name	Function
SLC25A40	Solute Carrier Family 25 Member 40	Probable mitochondrial transporter required for glutathione import into mitochondria, Glutathione, which plays key roles in oxidative metabolism, is produced exclusively in the cytosol and is imported in many organelles.
SOD1	Superoxide Dismutase 1	Destroys radicals which are normally produced within the cells and which are toxic to biological systems.
SOD2	Superoxide Dismutase 2	Destroys superoxide anion radicals which are normally produced within the cells and which are toxic to biological systems.
SOD3	Superoxide Dismutase 3	Protects the extracellular space from toxic effect of reactive oxygen intermediates by converting superoxide radicals into hydrogen peroxide and oxygen.
SRXN1	Sulfiredoxin 1	Oxidative stress resistance by reducing cysteine-sulfinic acid formed under exposure to oxidants.
STIP1	Stress-induced phosphor-protein 1	Co-chaperone for HSP90AA1. Mediates the association of the chaperones HSPA8/HSC70 and HSP90.
TXN	Thioredoxin	Redox reactions through oxidation of its dithiol to disulfide and dithiol-disulfide exchange reactions.
TXN2	Thioredoxin 2	Control of mitochondrial ROS homeostasis, apoptosis regulation and cell viability. Dithiol-reducing activity.
TXNDC2	Thioredoxin domain containing 2	Reduces disulfide bonds in the presence of NADP and TXN reductase.
TXNDC5	Thioredoxin domain containing 5	Protein disulfide isomerase of the ER forming disulfide bonds in proteins.
TXNDC8	Thioredoxin domain containing 8	Post-translational protein modifications required for acrosomal biogenesis.
TXNDC9	Thioredoxin domain containing 9	Diminishes chaperonin TCP1 complex ATPase activity, thus negatively impacts protein folding.
TXNDC11	Thioredoxin domain containing 11	Redox regulator of DUOX protein folding.
TXNDC12	Thioredoxin domain containing 12	Protein-disulfide reductase of the ER forming disulfide bonds through its thiol-disulfide oxidase activity.
TXNDC15	Thioredoxin domain containing 15	Regulating ciliary hedgehog signaling.
TXNDC16	Thioredoxin domain containing 16	Protein binding.

**Symbol**	**Name**	**Function**
TXNDC17	Thioredoxin domain containing 17	Dithiol-disulfide exchange reactions. Elimination of hydrogen peroxide.
TXNIP	Thioredoxin-interacting protein	Oxidative stress mediator by inhibiting TXN activity.
TXNL1	Thioredoxin-like 1	Active TXN with redox potential.
TXNL4A	Thioredoxin-like 4A	Role in re-mRNA splicing as component of U5 snRNP and U4/U6-U5 tri-snRNP complexes.
TXNL4B	Thioredoxin-like 4B	Required in cell cycle progression for S/G_2 transition
TXNRD1	Thioredoxin reductase 1	Reducing H_2O_2. Regulating redox reactions, growth and differentiation.
TXNRD2	Thioredoxin reductase 2	Control of ROS levels and regulation of mitochondrial redox homeostasis.
TXNRD3	Thioredoxin reductase 3	Glutaredoxin and glutathione reductase.
